# Medium-term intraocular tamponade with perfluorocarbon liquid in vitrectomy for complex retinal diseases: a systematic review

**DOI:** 10.1186/s40942-026-00814-5

**Published:** 2026-02-07

**Authors:** Nobutaka Tachibana, Kosuke Aonuma, Mikihiro Shimizu, Maki Suzuki, Masahiro Ito, Masakazu Takayama, Hiroki Kaneko

**Affiliations:** 1 Department of Ophthalmology, Hamamatsu University School of Medicine, Hamamatsu, Japan; 2 Center for Clinical Research, Hamamatsu University Hospital, Hamamatsu, Japan

## Abstract

**Purpose:**

Perfluorocarbon liquid (PFCL) tamponade is increasingly used in vitrectomy for complex retinal disorders. However, its effectiveness, indications, and safety remain uncertain. This study aimed to systematically evaluate the clinical outcomes, safety, and indications of medium-term PFCL tamponade in vitrectomy for complex retinal disorders based on the existing literature.

**Methods:**

A systematic review was conducted following PRISMA 2020 guidelines. PubMed, Google Scholar, and Web of Science were searched for English-language studies (2005‒2025) reporting vitrectomy with postoperative medium-term PFCL tamponade for retinal detachment (RD) or open globe injury. Case reports, conference abstracts, commentaries, non-English publications, studies involving pediatric populations, studies with ≤ 10 eyes, and studies with potentially overlapping patient cohorts were excluded. Study quality was assessed using the Joanna Briggs Institute (JBI) checklist. Random-effects single-arm meta-analyses were applied for redetachment rates and visual improvement rates.

**Results:**

Seventeen studies comprising 775 eyes were included. Substantial heterogeneity was observed. The pooled redetachment and visual improvement rates were 8.49% and 74.91%, respectively. Common complications included cataract progression, elevated intraocular pressure, transient ocular hypertension, and deposit formation. However, most included studies were retrospective case series, and heterogeneity in study design and outcome definitions limited the interpretation of the pooled results.

**Conclusions:**

Medium-term PFCL tamponade may be a viable alternative or adjunct to silicone oil in selected complex RD cases, with favorable anatomical and visual outcomes. However, evidence is limited by retrospective designs, lack of control groups, heterogeneity, and biases. Further prospective comparative trials are necessary to validate these roles in clinical practice.

**Supplementary Information:**

The online version contains supplementary material available at 10.1186/s40942-026-00814-5.

## Introduction

Pars plana vitrectomy is widely conducted to treat complex retinal disorders such as rhegmatogenous retinal detachment (RRD) and open globe injuries (OGI). Perfluorocarbon liquids (PFCL) are frequently utilized as intraoperative adjuncts to stabilize and flatten the retina because of their high specific gravity, which helps in displacing the subretinal fluid and hemorrhage. PFCL is especially effective in cases of giant retinal tears (GRT), inferior breaks, and complicated retinal detachments (RD) involving proliferative vitreoretinopathy (PVR) [1, 2]. 

However, long-term intraocular tamponade with PFCL is reportedly linked to several complications, including corneal toxicity, retinal infiltration, and inflammatory reaction [3]. In response to these risks, a method called short- to medium-term PFCL tamponade has gained traction, in which PFCL is postoperatively retained in the eye for a limited period without the simultaneous utilization of silicone oil. This approach aims to achieve temporary retinal support while potentially decreasing the requirement for additional surgeries and simplifying surgical management.

Despite the increasing interest, evidence regarding the clinical efficacy, indications, and safety of medium-term PFCL tamponade remains limited. A comprehensive synthesis of outcomes, including anatomical success, redetachment rates, visual improvement, and complications, has not yet been performed.

This systematic review, therefore, aimed to assess the current evidence regarding vitrectomy using medium-term PFCL tamponade, focusing on surgical outcomes, retinal redetachment, visual improvement, and associated complications, to evaluate clinical outcomes and safety.

## Methods

This systematic review was conducted according to the Preferred Reporting Items for Systematic Reviews and Meta-Analyses (PRISMA) statement [4]. A systematic literature search was conducted using PubMed, Google Scholar, and Web of Science, covering publications between January 2005 and December 2025 (based on availability at the time of the search). The following search strategy was applied: (“short-term PFCL” OR “medium-term PFCL” OR “medium-term perfluoro-n-octane” OR “1-week PFCL” OR “perfluorocarbon liquid tamponade” OR “perfluoro-n-octane retention”) AND (“retinal detachment” OR “rhegmatogenous retinal detachment” OR “proliferative vitreoretinopathy” OR “giant retinal tear” OR “complex retinal detachment”) AND (“pars plana vitrectomy” OR “PPV” OR “vitrectomy”) AND (“retinal reattachment” OR “visual outcome” OR “anatomical success” OR “complications” OR “retinal toxicity”). Table 1 shows the detailed search strategies. All authors screened the titles and abstracts and subsequently reviewed the full texts to assess eligibility.

**Table 1:** Development of the final search strategy

Step	Search Component	Keywords / Phrases Used	Rationale
1	Intervention	“short-term PFCL”, “medium-term PFCL”, “1-week PFCL”, “medium-term perfluoro-n-octane”, “perfluorocarbon liquid tamponade”, “perfluoro-n-octane retention”	To capture various terminologies referring to short- or medium-term retention of perfluorocarbon liquid (PFCL)
2	Condition / Disease	“retinal detachment”, “rhegmatogenous retinal detachment”, “proliferative vitreoretinopathy”, “giant retinal tear”, “complex retinal detachment”	To cover the broad spectrum of retinal detachment conditions relevant to PFCL tamponade
3	Procedure	“pars plana vitrectomy”, “PPV”, “vitrectomy”	To ensure inclusion of studies involving vitrectomy as the surgical intervention
4	Outcomes	“retinal reattachment”, “visual outcome”, “anatomical success”, “complications”, “retinal toxicity”	To identify studies reporting clinical and anatomical outcomes relevant to PFCL tamponade

Studies were included if they met the following criteria: involved vitrectomy for RD or OGI, and included postoperative medium-term PFCL tamponade. The exclusion criteria were as follows: case reports, conference abstracts, commentaries, non-English language publications, studies published before 2005, studies involving pediatric populations, studies with ≤ 10 eyes, and studies with potentially overlapping cases. Data extracted included author names, year of publication, number of cases, retinal redetachment rate, visual outcome, and complications. Two reviewers independently extracted the data.

Risk of bias was assessed using the Joanna Briggs Institute (JBI) Critical Appraisal Checklist for Case Series. Although several included studies had comparative designs, all were appraised as case series to ensure methodological consistency, focusing on the clarity of case definition, inclusion criteria, and outcome reporting rather than causal inference.

Statistical and pooled analyses were performed using Python, a general-purpose programming language (version 3.11.5). Data processing was conducted using pandas; statistical analyses were primarily performed using statsmodels, with additional statistical calculations and numerical computations using SciPy. Figures were generated using matplotlib. A single-arm meta-analysis was performed using a random-effects model to pool binary outcomes, including redetachment and visual improvement rates, with between-study variance estimated using the DerSimonian–Laird method. For visual improvement rates, a logit transformation with continuity correction (0.5) was applied to account for studies reporting 0% or 100% events. Heterogeneity was assessed using Cochran’s Q statistic and the I² metric.

Ethics approval and consent to participate because this study is a systematic review of previously published literature and did not involve any new human participants or the collection of identifiable personal data. This review was registered in PROSPERO (CRD420251270427).

## Results

**Fig. 1:**
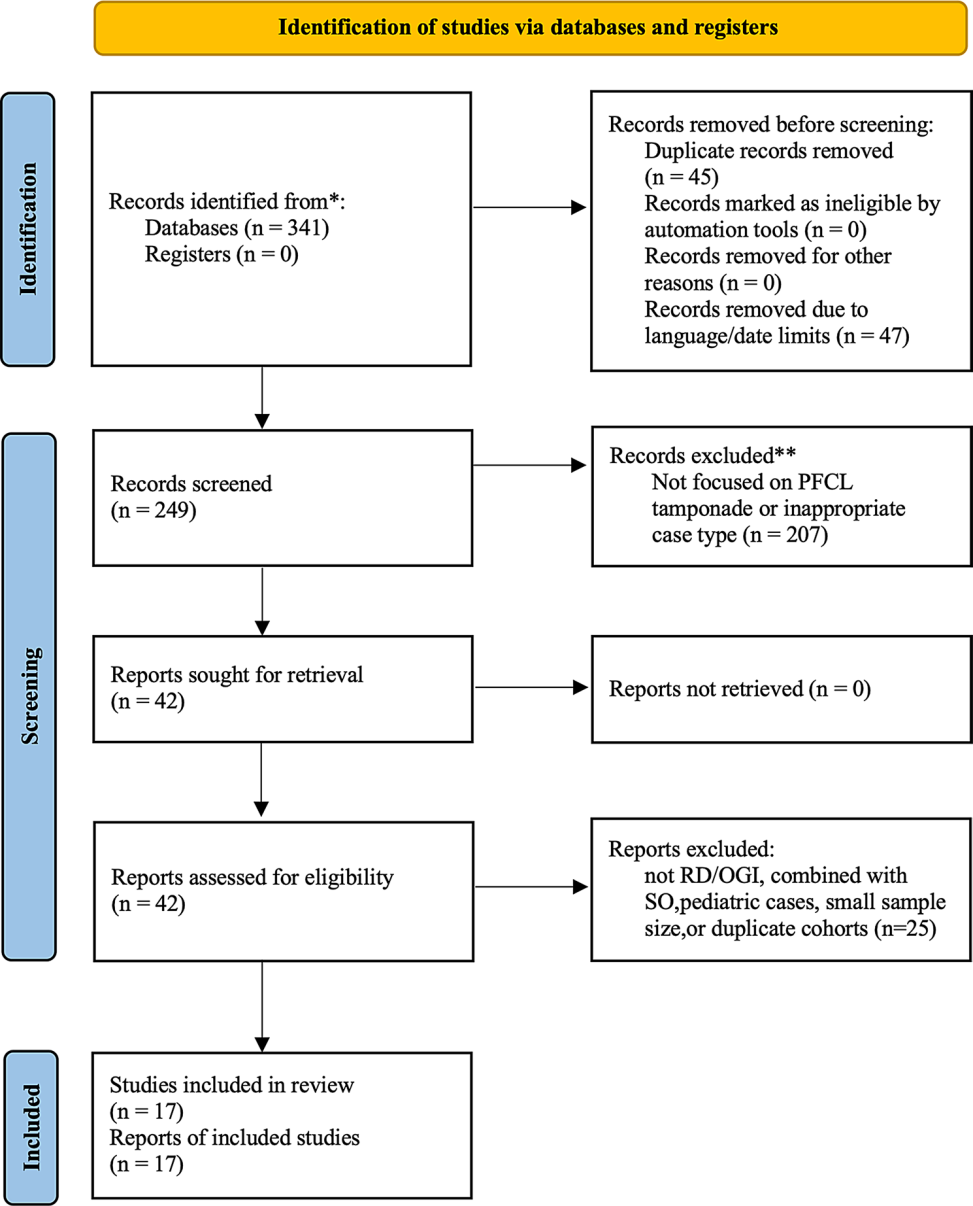
Flow diagram of study selection according to the PRISMA 2020 guidelines. Database searching in PubMed, Google Scholar, and Web of Science identified 341 records. 17 studies met the inclusion criteria and were included in the review. PFCL = perfluorocarbon liquid; RD = retinal detachment; OGI = open globe injury; SO = silicone oil

A Preferred Reporting Items for Systematic Reviews and Meta-Analyses (PRISMA) flow diagram (Fig. 1) illustrates the study selection process, and Supplementary Table 1 presents the PRISMA checklist. Database searches of PubMed, Google Scholar, and Web of Science identified 341 records. After removing duplicates, 296 records were screened. Forty-seven records were excluded owing to language and date mismatches, leaving 249 for title and abstract screening, of which 207 were excluded because they did not address PFCL tamponade or were case reports, conference abstracts, or commentaries. Forty-two full-text articles were assessed for eligibility, and 25 were excluded because they did not involve RD or OGI, involved combined use of silicone oil (SO), included pediatric populations, had ≤ 10 eyes, or potentially included overlapping cases. Ultimately, 17 studies met the inclusion criteria and were included in the review.

Table 2 shows the risk of bias for all 17 studies assessed using the JBI Critical Appraisal Checklist for Case Series (8 items). Clear inclusion criteria (Q1) and valid methods for condition identification (Q3) were consistently reported. In all studies, outcomes were evaluated using standardized and reliable clinical measures (Q2), including visual acuity, retinal reattachment status, intraocular pressure, and postoperative complications. In addition, demographic characteristics (Q6) and clinical information (Q7) were adequately described in all studies, allowing appropriate interpretation of patient backgrounds and disease severity. Conversely, the main potential sources of bias were related to the case inclusion process. Specifically, several studies did not clearly report whether cases were consecutively included (Q4) or whether all eligible cases during the study period were completely included (Q5). Consequently, these items were rated as having “moderate concern,” reflecting the possibility of selection bias due to insufficient reporting rather than inherent methodological flaws. Reporting of outcomes and follow-up (Q8) was generally adequate, with most studies clearly describing anatomical success rates, visual outcomes, postoperative complications, and follow-up duration. Overall, no study was judged to be at high risk of bias according to the JBI case series checklist. Two studies were rated as low risk across all eight items, while the remaining studies exhibited low to moderate risk of bias, primarily due to unclear reporting regarding consecutive case inclusion and completeness of case enrollment.

**Table 2:** Risk of bias assessment using the JBI critical appraisal checklist for case series

Author (Year)	1. Clear inclusion criteria	2. Standard, reliable measurement of condition	3. Valid identification of the condition	4.Consecutive inclusion	5.Complete inclusion	6. Reporting of demographics	7. Reporting of clinical information	8. Reporting of outcomes / follow-up	Over all
Citirik M et al. (2024) [5]	Low	Low	Low	Moderate	Moderate	Low	Low	Low	Moderate
Shukla D et al. (2023) [6]	Low	Low	Low	Low	Low	Low	Low	Low	Low
Naz S et al. (2022) [7]	Low	Low	Low	Moderate	Moderate	Low	Low	Low	Moderate
Keller J et al. (2021) [8]	Low	Low	Low	Moderate	Moderate	Low	Low	Low	Moderate
Churashov SV et al. (2021) [9]	Low	Low	Low	Moderate	Moderate	Low	Low	Low	Moderate
Chehade et al. (2021) [10]	Low	Low	Low	Low	Moderate	Low	Low	Low	Low
Bhurayanontachai et al. (2020) [11]	Low	Low	Low	Low	Moderate	Low	Low	Low	Low
Sheridan et al. (2019) [12]	Low	Low	Low	Low	Moderate	Low	Low	Low	Moderate
Zhang et al. (2018) [13]	Low	Low	Low	Low	Moderate	Low	Low	Low	Low
Mikhail MA et al. (2017) [14]	Low	Low	Low	Low	Moderate	Low	Low	Low	Moderate
Eiger-Moscovich M et al. (2017) [15]	Low	Low	Low	Low	Moderate	Low	Low	Low	Moderate
Randolph JC et al. (2016) [16]	Low	Low	Low	Low	Moderate	Low	Low	Low	Low
Sigler EJ et al. (2013) [17]	Low	Low	Low	Low	Low	Low	Low	Low	Low
Rush R et al. (2012) [2]	Low	Low	Low	Low	Moderate	Low	Low	Low	Low
Drury B et al. (2011) [18]	Low	Low	Low	Moderate	Moderate	Low	Low	Low	Moderate
Rofail M et al. (2005) [19]	Low	Low	Low	Low	Moderate	Low	Low	Low	Moderate
Sirimaharaj M et al. (2005) [20]	Low	Low	Low	Low	Moderate	Low	Low	Low	Moderate

In total, 775 eyes from the included 17 studies. Table 3 shows the characteristics of the included studies.

**Table 3:** Included studies of medium-term intraocular tamponade with PFCL

Authors	Year	study type	Sample Size	Retinal Redetachment *N*(eyes)	VA improved(≧baseline) *N*(eyes)		Complications *N*(eyes)
Citirik M et al. [5]	2024	Retrospective case-control study	48	3	−		Inflammation in the AC (7 eyes)
Shukla D et al. [6]	2023	Retrospective case series	35	2	30		Precipitates on retina and posterior capsule (8 eyes) Elevated IOP during PFCL placement (4 eyes), Hypotony (4 eyes) PFCL in the AC (3 eyes), Keratopathy (2 eyes) Small subretinal PFCL bubbles (1 eye), Inflammation in the AC (1 eye)
Naz S et al. [7]	2022	Retrospective case series	40	1	40		Cataract (4 eyes), Uveitis (2 eyes) Optic atrophy (2 eyes), Endophthalmitis (1 eye)
Keller J et al. [8]	2021	Retrospective case-control study	18	1	−		Elevated IOP during PFCL placement (8 eyes) ERM (4 eyes), CME (4 eyes), Retained PFCL (1 eye) Retinal translocation (1 eye)
Churashov SV et al. [9]	2021	Retrospective cohort study	20	9	−		−
Chehade et al. [10]	2021	Retrospective case series	85	1	−		Cataract (20 eyes), CME (14 eyes) PFCL in the AC (8 eyes) Elevated IOP after PFCL removal (3 eyes)
Bhurayanontachai et al. [11]	2020	Retrospective case series	122	24	102		Elevated IOP during PFCL placement (62 eyes) Elevated IOP after PFCL removal (31 eyes), Hypotony (28 eyes) Optic disc atrophy (27 eyes), Cataract (27 eyes), ERM (16 eyes)
Sheridan et al. [12]	2019	Retrospective case series	25	2	18		−
Zhang et al. [13]	2018	Retrospective case series	23	0	15		Posterior capsule opacity (16 eyes), Cataract (11 eyes) Precipitates on posterior capsule (7 eyes) Elevated IOP during PFCL placement (5 eyes) Conjunctival congestion (5 eyes) Keratic precipitates (3 eyes), Precipitates on retina (3 eyes)
Mikhail MA et al. [14]	2017	Retrospective case series	30	3	21		Cataract (15 eyes), Uveitis (6 eyes) Elevated IOP after PFCL removal (2 eyes) PFCL in the AC (1 eye)
Eiger-Moscovich M et al. [15]	2017	Retrospective case series	13	0	9		Elevated IOP after PFCL removal (2 eyes), Cataract (2 eyes) CME (2 eyes), PFCL in the AC (1 eye)
Randolph JC et al. [16]	2016	Retrospective case series	23	5	−		Cataract (10 eyes), Elevated IOP after PFCL removal (8 eyes) Precipitates on retina and posterior capsule (7 eyes)
Sigler EJ et al. [17]	2013	Retrospective case series	159	21	−		Elevated IOP after PFCL removal (54 eyes) Precipitates on retina and posterior capsule (43 eyes) PFCL in the AC (34 eyes), Cataract (22 eyes)
Rush R et al. [2]	2012	Retrospective case series	39	3	−		Posterior capsule opacity (28 eyes), Cataract (21 eyes) Elevated IOP during PFCL placement (14 eyes) PFCL in the AC (8 eyes), Inflammation in the AC (8 eyes), ERM (7 eyes)
Drury B et al. [18]	2011	Retrospective case series	17	4	14		Cataract (6 eyes), ERM (6 eyes), Corneal defect (4 eyes) Elevated IOP after PFCL removal (4 eyes) Retained PFCL (4 eyes), Posterior synechia (2 eyes) Small macula haemorrhage (1 eye), Atrophic macula (1 eye) Inflammation in the AC (1 eye)
Rofail M et al. [19]	2005	Retrospective case series	16	1	13		Cataract (6 eyes), ERM (4 eyes), Hypotomy (3 eyes) Keratopathy (1 eye), Inflammation in the AC (1 eye)
Sirimaharaj M et al. [20]	2005	Retrospective case series	62	4	34		Cataract (29 eyes), Glaucoma (3 eyes)

PFCL=Perfluorocarbon liquid, VA=Visual acuity, AC=Anterior chamber, IOP=Intraocular pressure, ERM=Epiretinal membrane, CME=Cystoid macular edema

The results of the pooled analyses for retinal redetachment rate and visual improvement rate using a random-effects model are presented in Table 4. Substantial between-study heterogeneity was observed for the redetachment rate, with Cochran’s Q = 42.65 (df = 16, *p* < 0.001) and I² = 62.5%. Similarly, moderate to high heterogeneity was observed for the visual improvement rate, with Cochran’s Q = 27.14 (df = 9, *p* = 0.0013) and I² = 66.8%.

**Table 4:** Heterogeneity statistics for retinal re-detachment and visual improvement rates among the included studies

Outcome	I² (Heterogeneity)	Cochran’s Q Statistic	Degrees of Freedom (df)	*p*-value (Q test)
Retinal Re-detachment Rate	62.50%	42.65	16	< 0.001
Visual Improvement Rate	66.80%	27.14	9	0.0013

**Fig. 2:**
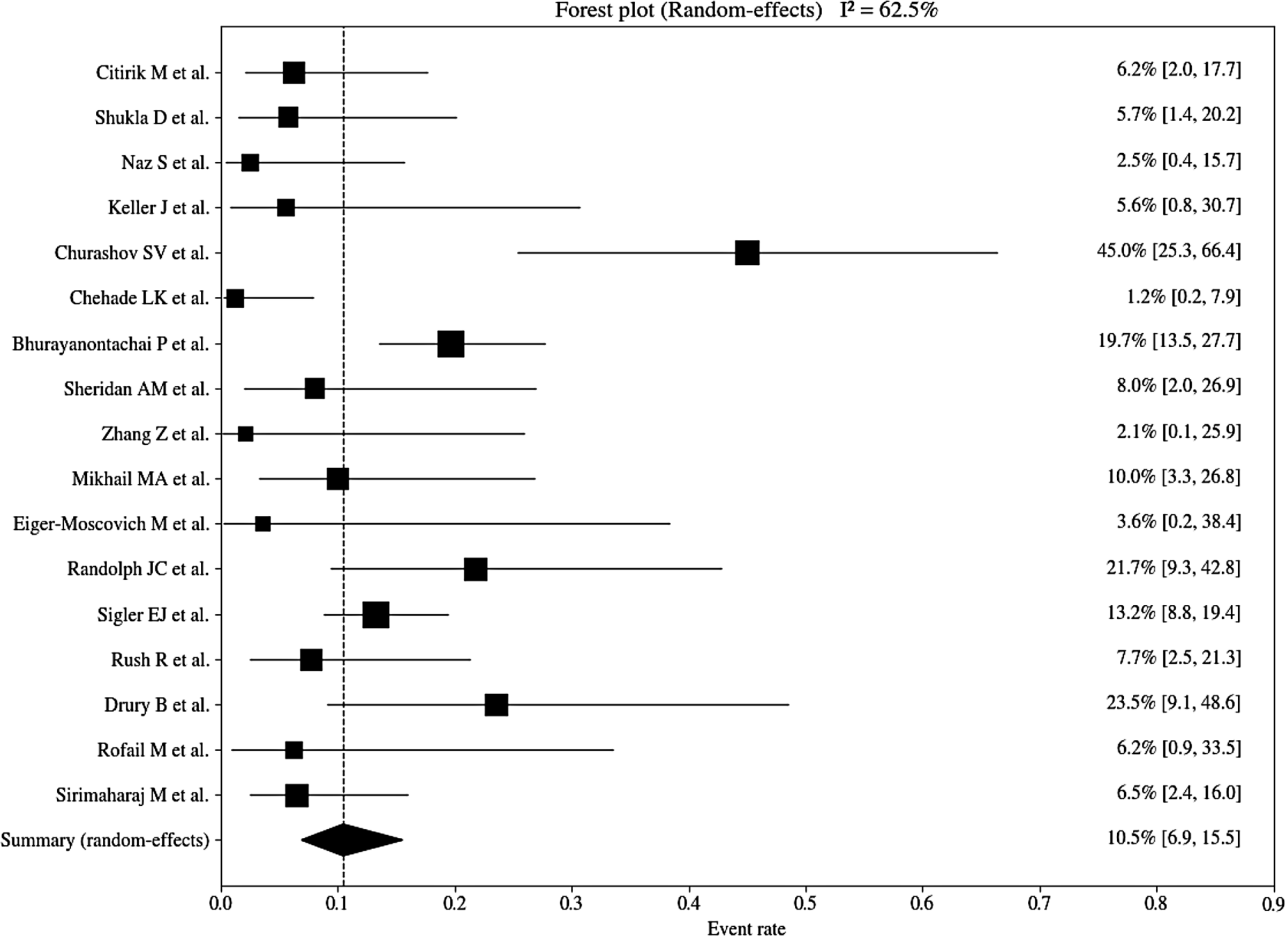
Forest plot of the redetachment rate after medium-term intraocular tamponade with PFCL. Event rates of retinal redetachment for individual studies are shown with 95% confidence intervals (CIs) (horizontal lines). Square markers represent the point estimates, with size proportional to the study weight. The black diamond depicts the pooled event rate, 10.5% (95% CI, 6.9%–15.5%). The vertical dashed line indicates the pooled estimate. The wider confidence intervals reflect smaller sample sizes. PFCL = perfluorocarbon liquid

**Fig. 3:**
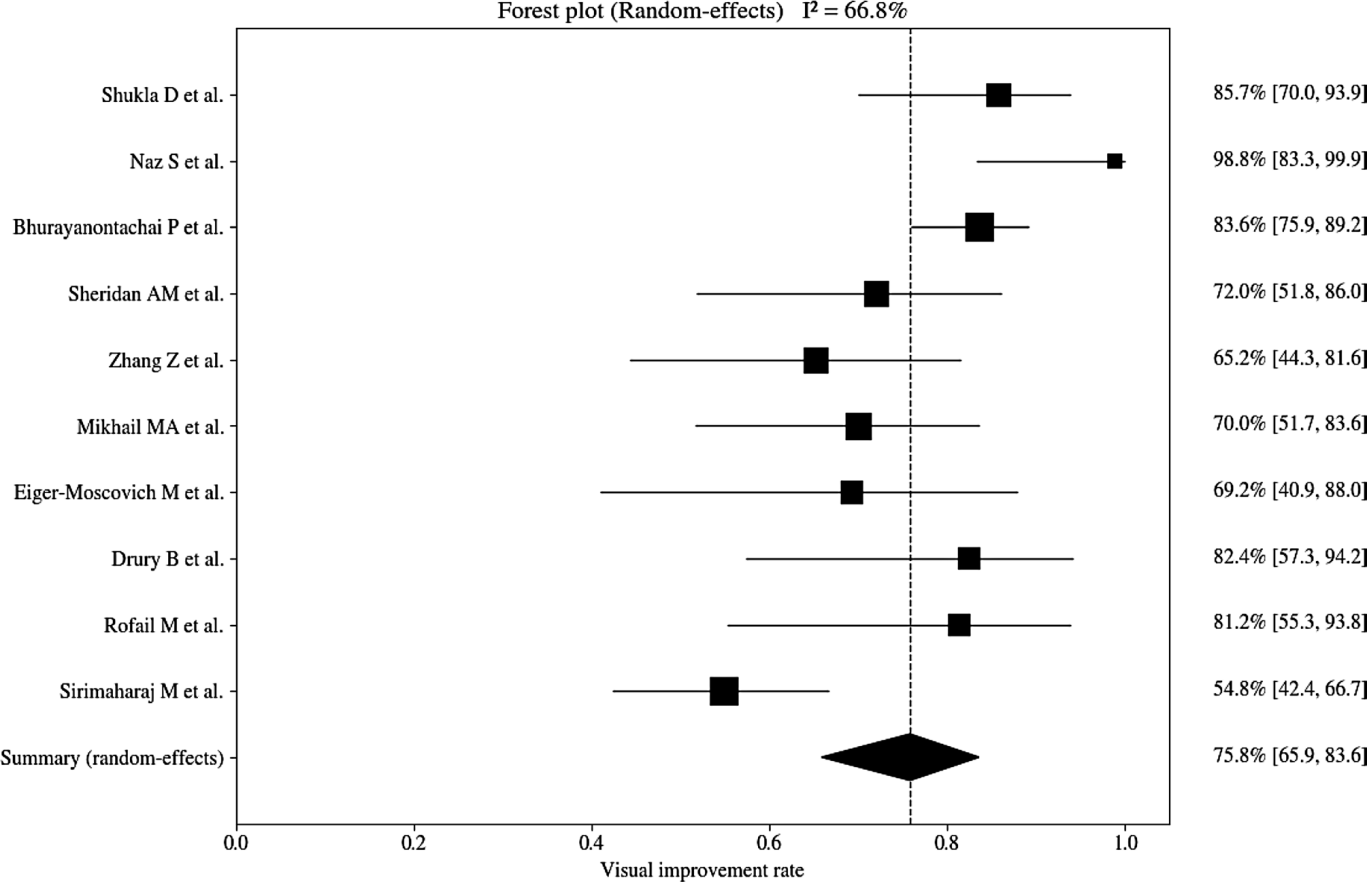
Forest plot of visual improvement after medium-term intraocular tamponade with PFCL. Proportions of visual improvement reported by individual studies are displayed with 95% confidence intervals (CIs) (horizontal lines). Square markers indicate study-specific point estimates, scaled by inverse-variance weight. The black diamond shows the pooled proportion under a random-effects model: 75.8% (95% CI, 65.9%– 83.6%). The vertical dashed line marks the pooled estimate; wider CIs reflect smaller sample sizes. PFCL, perfluorocarbon liquid

The pooled event rate for retinal redetachment was 10.5% using a random-effects model (95% confidence interval: 6.9‒15.5%; Fig. 2). Pooled analysis of visual improvement demonstrated an overall visual improvement rate of 75.8% based on a random-effects model (95% confidence interval: 65.9‒83.6%; Fig. 3).

Among the 775 eyes included across the analyzed studies, the most common complication was cataract progression, observed in 173 eyes (22.3%), followed by persistent intraocular pressure elevation or glaucoma after PFCL removal in 107 eyes (13.8%), transient intraocular pressure elevation during PFCL retention in 93 eyes (12.0%), precipitates on the posterior capsule or retina in 67 eyes (8.7%), PFCL migration into the anterior chamber in 55 eyes (7.1%), posterior capsule opacification in 44 eyes (5.7%), epiretinal membrane formation in 37 eyes (4.8%), hypotony in 35 eyes (4.5%), optic nerve atrophy in 29 eyes (3.7%), anterior chamber inflammation or iritis in 26 eyes (3.4%), cystoid macular edema in 20 eyes (2.6%), corneal degeneration or keratopathy in 7 eyes (0.9%), conjunctival congestion in 5 eyes (0.7%), residual PFCL in 5 eyes (0.65%), keratic precipitates in 3 eyes (0.4%), and posterior synechiae in 2 eyes (0.3%).

## Discussion

RRD with inferior retinal breaks or PVR, as well as ocular trauma, are clinical conditions prone to poor postoperative outcomes. Rush et al. noted that in GRT cases, multiple breaks involving several quadrants, or RD with inferior breaks, postoperative tamponade with SO or gas may be insufficient or biased toward the superior retina, potentially leading to redetachment [2]. In particular, if strict prone positioning cannot be maintained, fluid may enter through the retinal breaks before adequate chorioretinal adhesion induced by laser photocoagulation or cryotherapy is achieved, resulting in reopening of the breaks. However, maintaining a prone position postoperatively is difficult for many patients. Conversely, the supine position is relatively easier to maintain; therefore, PFO and other PFCLs may offer an advantage over SO or gas tamponade, especially in cases involving inferior retinal breaks.

PFCLs were first introduced for vitreoretinal surgery by Chang et al. in 1988 [21]. They have been shown to stabilize the retina and reduce traction during membrane peeling in eyes with PVR, as well as to unfold and flatten the retina in cases of GRT [22]. Blinder et al. were the first to investigate PFCLs as a short-term postoperative tamponade, demonstrating their safety and efficacy [23].

Residual PFCL within the vitreous cavity is a well-recognized cause of complications such as intraocular inflammation and sustained intraocular pressure elevation. In addition, PFCL-associated morphological changes and potential retinal toxicity represent important safety concerns [5, 24, 25]. Srivastava et al. reviewed recently reported cases of PFCL-associated acute retinal toxicity, particularly PFO, and highlighted the limitations of conventional quality control systems [26]. Although PFCLs have long been considered biologically inert, evidence suggests that observed toxicity is attributable to impurities introduced or generated during manufacturing and storage, resulting in substantial variability between production lots. Notably, traditional indirect cytotoxicity assays may underestimate PFCL toxicity and yield false-negative results because of the hydrophobic and volatile properties of these substances. Accordingly, the authors advocated that each PFCL lot should undergo chemical analysis in addition to direct-contact cytotoxicity testing using viable cells, providing a theoretical basis for the strengthened safety requirements outlined in ISO 16672:2020. Rush et al. further emphasized the importance of using PFO as a short-term postoperative tamponade and exchanging it for gas or SO within 10 days. As PFCL migration into the anterior chamber is associated with elevated intraocular pressure, anterior chamber paracentesis and thorough irrigation were performed at the time of PFCL removal. The authors further stressed the need for careful monitoring of intraocular pressure and intraocular inflammation during PFCL retention [2]. Experimental studies have demonstrated that PFCLs show relatively good tolerability with short-term retinal contact, whereas prolonged intraocular retention may induce time- and gravity-dependent retinal damage. In a rabbit eye model reported by Eckardt et al., no obvious retinal damage was observed after short-term PFCL exposure for 8 h; however, progressive histopathological changes were noted with retention periods ranging from 6 days to 2 months, predominantly affecting the inferior retina. The principal findings included marked Müller cell hypertrophy with protrusion beyond the external limiting membrane, accompanied by loss of photoreceptor outer segments, a reduction in the number of cells in the granular layer, thinning of the outer plexiform layer, and progressive hypertrophy of the retinal pigment epithelium with drusen formation [27]. These changes appear to be independent of PFCL type and are more likely attributable to physical factors, such as sustained contact related to the high specific gravity of the substance, rather than intrinsic chemical toxicity. The localization of lesions to the inferior retina, which remained in continuous contact with PFCL, further suggests that gravity-dependent alterations in the local microenvironment and mechanical stress play a more important role than direct toxic effects. These experimental findings support the notion that PFCLs are unsuitable as long-term vitreous substitutes and simultaneously validate the current clinical strategy of restricting PFCL use to intraoperative application or short-term postoperative tamponade. When the retention period is strictly controlled and careful postoperative monitoring is ensured, PFCL may serve as a valuable adjunct in the management of complex RD.

In this systematic review and pooled analysis using a random-effects model, the retinal redetachment rate was 10.5%, and the visual improvement rate was 75.8% in RD surgery employing short- to medium-term PFCL tamponade. This approach showed low redetachment rates, high visual improvement rates, and an acceptable safety profile. Short- to medium-term PFCL tamponade appears promising compared with long-term SO tamponade, potentially providing faster visual recovery.

Substantial heterogeneity was observed across several outcome categories. For anatomical success and redetachment rates, the primary sources of heterogeneity included differences in underlying retinal pathologies (such as GRTs, PVR, and ocular trauma), surgical indications, and the duration of postoperative PFCL tamponade. Secondary sources included variations in adjunctive surgical procedures, PFCL types, and follow-up periods. Regarding visual improvement, primary heterogeneity arose from differences in baseline visual acuity, macular status, and outcome definitions across studies, while secondary factors included lens status, postoperative cataract progression, and coexisting retinal or optic nerve pathology.

Regarding safety, the overall analysis of 775 eyes demonstrated that cataract progression and intraocular pressure elevation or glaucoma were the most frequently observed complications. These adverse events are commonly reported after vitreoretinal surgery and with heavy tamponade agents, and do not strongly suggest severe PFCL-specific toxicity. Nevertheless, PFCL-related complications such as anterior chamber migration, foreign body reactions, and retinal precipitates were observed in a subset of cases, underscoring the importance of strict control of the retention period and timely removal of PFCL.

A major strength of this review lies in its focus on short- to medium-term PFCL tamponade and its integrated evaluation of clinically relevant outcomes, namely retinal redetachment rate and visual improvement rate. However, some limitations should be acknowledged. Most of the included studies were retrospective observational studies or case series and lacked control groups. In addition, there was substantial heterogeneity in outcome definitions and PFCL retention durations, and the potential influence of selection bias and reporting bias could not be fully excluded.

Therefore, although based on limited clinical evidence, short- to medium-term PFCL tamponade appears to offer effective anatomical support with relatively favorable functional outcomes. However, current data remain insufficient, and the associated risks have not been fully elucidated. Nonetheless, in carefully selected cases—particularly those requiring inferior tamponade or staged surgical repair—PFCL may serve as an alternative or adjunct to SO. Given the limited number of studies and heterogeneity in study design, the findings of this review and pooled analysis should be interpreted with caution. Further validation through high-quality prospective studies and randomized controlled trials is essential to clarify the role of PFCL tamponade in vitreoretinal surgery.

## Supplementary Information

Below is the link to the electronic supplementary material.

Supplementary Material 1

## Data Availability

This review was registered in PROSPERO (CRD420251270427).
